# Supplementary material for: Antimicrobial Usage and Resistance in Companion Animals: A Cross-Sectional Study in Three European Countries
Source: Antibiotics (Basel). 2020 Feb 16;9(2):87. doi: 10.3390/antibiotics9020087 (PMC7175148; doi:10.3390/antibiotics9020087)
Supplement: Supplementary file 1 [file antibiotics-09-00087-s001.pdf]

Supplementary materials

Antimicrobial usage and resistance in companion animals: a cross-sectional study in three European countries

Philip Joosten [1*], Daniela Ceccarelli [(2)†], Evelien Odent [(1)], Steven Sarrazin [(1)], Haitske Graveland [(3)], Liese Van Gompel [4], Antonio Battisti [5], Andrea Caprioli [5], Alessia Franco [5], [EFFORT group], Jaap A. Wagenaar [2,3], Dik Mevius [2,3], Jeroen Dewulf [1]

[1] Veterinary Epidemiology Unit, Department of Obstetrics, Reproduction and Herd Health, Faculty of veterinary Medicine, Ghent University, Salisburylaan 133, Merelbeke 9820, Belgium; philip.joosten@ugent.be, evelienodent@gmail.com, s.sarrazin@lammerant.be, jeroen.dewulf@ugent.be

[2] Department of Bacteriology and Epidemiology, Wageningen Bioveterinary Research, Lelystad, Netherlands; daniela.ceccarelli@wur.nl, J.Wagenaar@uu.nl, D.J.Mevius@uu.nl

[3] Department of Infectious Diseases and Immunology, Faculty of Veterinary Medicine, Utrecht University, Yalelaan 1, 3584 CL Utrecht, the Netherlands; haitskegraveland@hotmail.com, J.Wagenaar@uu.nl

[4] Institute for Risk Assessment Sciences, Utrecht University. Yalelaan 2, 3584 CM Utrecht, the Netherlands; L.VanGompel@uu.nl

[5] Istituto Zooprofilattico Sperimentale del Lazio e della Toscana "M. Aleandri", Department of General Diagnostics, National Reference Laboratory for Antimicrobial Resistance, Via Appia Nuova, 1411, 00178, Rome, Italy; antonio.battisti@izslt.it, andrea.caprioli@izslt.it, alessia.franco@izslt.it

* Correspondence: philip.joosten@ugent.be; Tel.: +32-(0)92647548

† Current Affiliation: Research Executive Agency, European Commission, Brussels, Belgium

(..) No longer related to the affiliation

Supplementary Materials Part A: univariate logistic regression to antimicrobial usage

To look for aspects influencing antimicrobial usage (AMU) in companion animals, factors were created based on the information retrieved from the questionnaires. These factors represent general information of the animal such as age, breed and sex, but also include information regarding contacts with other animals, its environment or diet. Not all questions were transformed into factors, due to a limited number of answers on questions that were not applicable for all animals or because of the irrelevance of the question. After creating the factors, they were used in a univariate logistic regression with AMU as binary dependent variable, meaning that this variable represents "if the animal had received an antimicrobial treatment during the study period, yes or no". Each factor was seen as an independent variable (binary, categorical, continuous) to look at the relation between this factor and AMU. Factors could extrapolated from questions that were included in both dog and cat questionnaires (further referred to as common factors) or from just one of them, when only relevant for one of the two species (further referred to as species-specific factors). Common factors were analyzed for the whole population and for each species separately. Species-specific factors were only analyzed for the relevant species. All factors are listed below in Table S1, only the significant ones (p-value < 0.05) are reported with the responding coefficient and *p*-value in the main text.

Table 1. Overview of factors used in a univariate logistic regression with antimicrobial usage as a binary dependent variable.

Factor[1]	Question number[2]	Question category[3]	Cats/dogs/ both[4]	Type of variable[5]	Significant cats/dogs/all[6]
Species	n/a	n/a	both	Cat - Dog	- / - / **yes**
Country	n/a	n/a	both	BE – NL - IT	**yes** /no /no
Live in residential area -	n/a	Section B	both	0-1	no / no / no
Live in rural area	n/a	Section B	both	0-1	no / no / no
Live in wooded area	n/a	Section B	both	0-1	no / no / no
Live in other area	n/a	Section B	both	0-1	no / no / no
Breed	n/a	Section C	both	0-1	no / no / no
Age	n/a	Section C	both	continuous	no / no / no
Sex	n/a	Section C	both	Male-female	no / no / no
Neutered	n/a	Section C	both	0-1	no / no / no
No contact with other animals	23	Section D	both	0-1	no / no / no
Contact with other companion animals	23	Section D	both	0-1	no / no / no
Contact with farm animals	23	Section D	both	0-1	no / no / no
Contact with both	23	Section D	both	0-1	no / no / no
N of animals in house 1	25	Section D	both	0-1	no / no / no
N of animals in house 2-5	25	Section D	both	0-1	no / no / no

N of animals in house >5	25	Section D	both	0-1	no / no / no
Stays always inside	24	Section D	both	0-1	no/ no/ **yes**
Stays more inside	24	Section D	both	0-1	no / no / no
Stays inside – outside 50%	24	Section D	both	0-1	no / no / no
Stay more outside	24	Section D	both	0-1	no / no / no
Stays always outside	24	Section D	both	0-1	no / no / no
Stayed in shelter	27	Section D	both	0-1	no / no / no
Nearby farm - horse	31-32	Section D	both	0-1	no / no / no
Nearby farm - cattle	31-32	Section D	both	0-1	no / no / no
Nearby farm - pigs	31-32	Section D	both	0-1	no / no / no
Nearby farm - poultry	31-32	Section D	both	0-1	no / no / no
Access to farm	33	Section D	both	0-1	no / no / no
Access to lake	34	Section D	both	0-1	no / no / no
Access to ditch	34	Section D	both	0-1	no / no / no
Access to toilet water	34	Section D	both	0-1	no / no / no
Access to other water	34	Section D	both	0-1	no / no / no
Eats dry food	35	Section E	both	0-1	no / no / no
Eats wet food	35	Section E	both	0-1	no / no / no
Eats cooked non commercial meat	35	Section E	both	0-1	no / no / no
Eats thawed raw meat	35	Section E	both	0-1	no / no / no
Eats raw meat	35	Section E	both	0-1	no / no / no
Eats vegetarian food	35	Section E	both	0-1	no / no / no
Eats leftovers	35	Section E	both	0-1	no / no / no
Eats other	35	Section E	both	0-1	no / no / no
Hospitalized <6M	39	Section F	both	0-1	no / no / no
Eats faeces	28	Section D	dogs	0-1	- / no / -
Dog walking	30	Section D	dogs	0-1	- / no / -
Off leash	30	Section D	dogs	0-1	- / no / -
Hunting	30	Section D	dogs	0-1	- / no / -
Stayed in dog day care	30	Section D	dogs	0-1	- / no / -
Goes to dog park	30	Section D	dogs	0-1	- / no / -
Does Agility	30	Section D	dogs	0-1	- / no / -
Eats dried pig ear	36	Section E	dogs	0-1	- / no / -

Eats raw bones	36	Section E	dogs	0-1	- / no / -
Eats cooked bones	36	Section E	dogs	0-1	- / no / -
Eats store bought	36	Section E	dogs	0-1	- / no / -
Eats rawhide chews	36	Section E	dogs	0-1	- / no / -
Eats other treats	36	Section E	dogs	0-1	- / no / -
Catch & eat prey	6	Section D	cats	0-1	no / - / -

[1]name of the created factor, [2]The question number relates to the number of the question in the questionnaire on which the factor was based, [3]the category relates to the category of the questionnaire to which the question belongs, [4]indicates if the question is part of one specific questionnaire (cats or dogs), or is part of both, [5]type of variable; binary (0-1), continuous, categorical (category 1 - category 2 - category 3), [6]indicates if the factor is significant in the univariate logistic regression for cats (cats / … / …), dogs (…/ dogs / …) and/or the total study population (… / … / all).

Supplementary Materials Part B: Results on antimicrobial usage (not shown in the main text)

Table 2. The total number of antimicrobial treatments per active substance in dogs and cats, stratified per country and in total.

	**BE**			**IT**			**NL**			**Total**		
	**Dogs (n[1]=12)**	**Cats (n[1] = 15)**	**Total (n[1] = 27)**	**Dogs (n[1] = 16)**	**Cats (n[1] = 4)**	**Total (n[1] =20)**	**Dogs (n[1] =27)**	**Cats (n[1] = 10)**	**Total (n[1] = 37)**	**Dogs (n[1] = 55)**	**Cats (n[1] =29)**	**ALL (n[1] = 84)**
Amoxicillin	-	2	**2**	2	-	**2**	2	1	**3**	4	3	**7**
Amoxicillin-clavulanate	1	4	**5**	5	1	**6**	9	3	**12**	15	8	**23**
Cefalexin	4	-	**4**	1	-	**1**	1	-	**1**	6	-	**6**
Cefazolin	1	-	**1**	-	-	**-**	-	-	**-**	1	-	**1**
Cefovecin	-	4	**4**	-	2	**2**	1	-	**1**	1	6	**7**
Chloramphenicol	-	-	**-**	-	-	**-**	2	1	**3**	2	1	**3**
Clindamycine	1	1	**2**	-	-	**-**	1	-	**1**	2	1	**3**
Doxycycline	-	1	**1**	1	-	**1**	1	1	**2**	2	2	**4**
Enrofloxacin	3	2	**5**	-	1	**1**	-	1	**1**	3	4	**7**
Gentamicin	1	-	**1**	-	-	**-**	-	-	**-**	1	-	**1**
Marbofloxacin	1	-	**1**	1	-	**1**	-	-	**-**	2	-	**2**
Metronidazole	-	-	**-**	2	-	**2**	-	-	**-**	2	-	**2**
Ofloxacine	-	1	**1**	-	-	**-**	-	-	**-**	-	1	**1**
Orbifloxacine	-	-	**-**	1	-	**1**	-	-	**-**	1	-	**1**
Penicillin	-	-	**-**	1	-	**1**	1	1	**2**	2	1	**3**
Polymyxin-b	-	-	**-**	-	-	**-**	3	2	**5**	3	2	**5**
Spiramycine-metronidazole	-	-	**-**	2	-	**2**	4	-	**4**	6	-	**6**
Trimethoprim-sulfonamide	-	-	**-**	-	-	**-**	2	-	**2**	2	-	**2**

[1.] **n represents the total number of treatments. BE = Belgium; IT = Italy; NL = the Netherlands.**

Supplementary Materials Part C: univariate logistic regression to antimicrobial resistance, expressed as multi drug resistance and full susceptibility.

To look for aspects influencing antimicrobial resistance (AMR) in companion animals, factors were created based on the information retrieved from the questionnaires such as species (dog vs cat) and country (Belgium vs Italy vs The Netherlands). After creating the factors, they were used in a univariate logistic regression with AMR as binary dependent variable. There are two different regressions as AMR is expressed by two different parameters. The first one being full susceptibility, meaning the isolate did not show resistance to any of the tested antimicrobials. The second one being multi drug resistance (MDR), meaning that the isolate showed resistance to any three or more antimicrobials from the tested panel. Each factor was seen as an independent variable, binary in the case of species, categorical in the case of country, to look at the relation between this factor and AMR. The results are listed below in Table S3, only when significant (p-value < 0.05), are 95% confidence intervals reported.

Table 3. Antimicrobial resistance expressed as full susceptibility (FS) or multi drug resistance (MDR) and influencing factors.

Country	Factor	Log. Regression OR[1] β – p-value	OR[1] [95% CI]	Study Population Total (%FS – %R)	Log. Regression OR[2] β – p-value	OR[2] [95% CI]	Study Population Total (%MDR)
**BE[a]**	Cats	-	-	44 (75% – 25%)	-	-	44 (9%)
	Dogs	-0.1 – 0.8	-	44 (80% – 20%)	-0.2 – 0.6	-	44 (11%)
	**Country**	**1.0 – 0.002**	**2.7 [1.4 – 5.1][a]**	**88 (77%– 23%)**	**-0.3 – 0.4**	**-**	**88 (10%)**
**IT[b]**	Cats	-	-	50 (48% – 52%)	-	-	50 (32%)
	Dogs	-0.9 – 0.03	0.4 [0.2-0.9]	50 (70% – 30%)	1.1 – 0.04	2.9 [1.1 – 8.3]	50 (14%)
	**Country**	**1.4 – <0.001**	**3.9 [2.0 – 7.8][b]**	**100 (59% – 41%)**	**-0.4 – 0.2**	**-**	**100 (23%)**
**NL[c]**	Cats	-	-	43 (91% – 9%)	-	-	43 (2%)
	Dogs	-0.1 – 0.7	-	51 (76% – 24%)	1.0 – 0.08	-	51 (8%)
	**Country**	**-0.4 – 0.3**	**-**	**94 (83% – 17%)**	**-0.2 – 0.6**	**-**	**94 (5%)**
**Total**	Cats	-	-	137 (70%– 30%)	-	-	137 (15%)
	Dogs	0.4 – 0.09	-	145 (75% – 25%)	-0.6 – 0.045	1.8 [1.0 – 3.3]	145 (11%)
	**Country**	**-**	**-**	**282 (73% – 27%)**	**-**	**-**	**282 (13%)**

[95% CI] = 95% Confidence Interval. BE = Belgium; IT = Italy; NL = The Netherlands; OR1 shows the odds ratio for species and country as risk factors for FS, on country level and overall (cats are used as reference); a,b,c comparison between two countries: [a]IT-BE, [b]IT-NL, [c]NL-BE (first country is used as reference); OR2 shows the odds ratio for species and country as a risk factor for MDR, on country level and overall (dogs are used as reference); a,b,c comparison between two countries: [a]IT-BE, [b]IT-NL, [c]NL-BE (first country is used as reference)

Supplementary Materials Part D: Results on antimicrobial resistance (not shown in the main text)

Table 4. Antimicrobial resistance against a set of 14 antimicrobials in commensal *E. coli* isolated from faeces of dogs and cats in three European countries (expressed as a percentage of the total number of isolates).

		AMP	FOT	TAZ	MERO	CIP	NAL	AZI	CHL	COL	GEN	SMX	TMP	TET	TGC	TOTAL[2]
BE	**Dogs (n[1]= 44)**	14%	0%	0%	0%	7%	5%	0%	2%	0%	0%	18%	11%	14%	0%	20%
	**Cats (n[1] = 44)**	18%	2%	2%	0%	2%	0%	0%	5%	0%	5%	11%	7%	7%	0%	25%
	**Total (n[1] = 88)**	18%	1%	1%	0%	5%	2%	0%	3%	0%	2%	15%	9%	10%	0%	23%
IT	**Dogs (n[1] = 50)**	14%	0%	0%	0%	12%	12%	2%	4%	0%	2%	18%	6%	14%	0%	30%
	**Cats (n[1] = 50)**	44%	6%	6%	0%	28%	24%	2%	8%	0%	2%	30%	18%	32%	0%	52%
	**Total (n[1] = 100)**	29%	3%	3%	0%	20%	18%	2%	6%	0%	2%	24%	12%	23%	0%	41%
NL	**Dogs (n[1] = 51)**	14%	0%	0%	0%	2%	0%	4%	0%	2%	0%	10%	10%	14%	0%	24%
	**Cats (n[1] = 43)**	5%	0%	0%	0%	0%	0%	0%	0%	2%	0%	2%	2%	2%	0%	9%
	**Total (n[1] = 94)**	10%	0%	0%	0%	1%	0%	2%	0%	2%	0%	6%	6%	9%	0%	17%
Total	**Dogs (n[1] = 145)**	14%	0%	0%	0%	7%	6%	2%	2%	1%	1%	15%	9%	14%	0%	25%
	**Cats (n[1] =137)**	23%	3%	3%	0%	11%	9%	1%	4%	1%	2%	15%	9%	15%	0%	30%
	**ALL (n[1] = 282)**	18%	1%	1%	0%	9%	7%	1%	3%	1%	1%	15%	9%	14%	0%	27%

[1]n represents the number of E. coli successfully isolated from faeces samples and tested by broth microdilution method. BE, Belgium; IT, Italy; NL, the Netherlands. AMP, ampicillin; FOT, cefotaxime; TAZ, ceftazidime; MERO, Meropenem; CIP, ciprofloxacin; NAL, nalidixic acid; AZI, azithromycin; CHL, chloramphenicol; COL, colistin; GEN, gentamicin; SMX, sulfamethoxazole; TMP, trimethoprim; TET, tetracycline; TGC, tigecycline.[2]TOTAL shows the percentage of isolates that were resistant to at least 1 antimicrobial.

Supplementary Materials Part E: Questionnaire Cat

I. General information

A. Administrative details veterinarian/veterinary practice

**Name + first name**	
**Address (personal)**	Street: nr.:
	Postal code: City:
	Country:
**Telephone nr.**	
**Mobile phone nr.**	
**Email address**	

A. Administrative details cat owner

**Name + first name**	
**Address (personal)**	Street: nr.:
	Postal code: City:
	Country:

**Description of residential area**	☐ Resident (mainly pavement and houses)<br>☐ Rural (many feedlot, little forestation)<br>☐ Wooded<br>☐ Other (please specify):..
**Telephone nr.**	
**Mobile phone nr.**	
**Email address:**	

General information Cat

**Name cat:**	
**Breed cat:**	
**Age**	
**Sex cat:**	☐ Female cat<br>☐ Male cat
**Neutered/sterilized**	☐ Yes<br>☐ No

A. Contact with other animals

1. Does your cat have contact with other animals, including farm animals?

☐ Yes, (please specify which animals)

☐ No

2. How many animals do you keep in house, including your cat?

☐ 1
☐ 2
☐ 3
☐ 4
☐ 5
☐ >5

3. Which animals do you keep in house?

....

4. How much time per week does your cat spend in the house or outside?

☐ Always in the house (100% inside)
☐ Mostly in the house , occasionally outside (80% inside, 20% outside)
☐ Often in the house, sometimes outside (60% inside, 40% outside)
☐ Half of the time in the house and half of the time outside (50% inside, 50% outside)
☐ Often outside, sometimes in the house (60% outside, 40% inside)
☐ Mostly outside, occasionally in the house (80% outside, 20% inside)
☐ Always outside (100% outside)
☐ Did your cat stay in a pension/animal shelter in the past year?

☐ No

☐ Yes

Period: From .... (dd/mm/yyyy) to .... (dd/mm/yyyy)

5. Does your cat catch and eat animals of prey?

☐ Yes

☐ No (go to question 9)

☐ Unknown (go to question 9)

6. Which kind of animals does your cat catch and eat prey?

....

7. At what frequency does your cat catch and eat prey?

☐ Weekly (or more)

☐ Monthly

☐ Lees than monthly

8. Are farms with livestock situated within a radius of 500 meters of your home?

☐ Yes

☐ No (go to question 12)

☐ Unknown (go to question 12)

9. Which type(s) of farm(s) are located within a radius of 500 meters of your home?

....

10. Does the cat have access to farms with livestock?

☐ Yes

What type(s) of farm(s) ....

....

☐ No

☐ Unknown

11. Has your cat had access to any of the following water sources, either for drinking or swimming, in the last six months? (Tick multiple boxes if applicable)

☐ Lakes, rivers, creeks

☐ Water in ditches, puddles

☐ Toilet

☐ Other, namely:

☐ No

☐ Unknown

A. Food

12. What does your cat eat? (Tick multiple boxes if applicable)

☐ Dry food (prepacked (commercial))

☐ Wet food (prepacked (commercial))

☐ Cooked non-commercial meat

☐ Defrozen raw meat

☐ Raw meat

☐ Vegetarian

☐ Prey

☐ Food remaining from the table

☐ Other, please specify:

B. Health

13. Is your cat currently healthy?

☐ Yes
☐ No: Please specify why you think your cat is not healthy:

....

....

14. Has your cat been treated with medicines in the last year?

Please ask your veterinarian for a printout of the medical records.

☐ Yes, please fill in the table below.
☐ No

Name of the medicine	Period of use<br>From (dd/mm/yyyy)<br>To (dd/mm/yyyy)	Frequency of use<br>Number of times per day	Duration of use<br>Number of days
1.			
2.			
3.			

4.			
5.			
6.			
7.			
8.			
9.			
10.			

15. Has your cat been hospitalized in the last six months?

☐ Yes
☐ No (go to question 19)

16. What was the reason for hospitalization?

....

17. During which period was the hospitalization?

From (dd/mm/yyyy) .... To (dd/mm/yyyy) ....

18. Do you have noticed something remarkable in the stool of your cat in the past three months?

☐ Yes
☐ No (go to question 21)

19. What have you noticed in the stool of your cat? (Tick multiple boxes if applicable)

☐ Reduced consistency
☐ Increased consistency
☐ Light colour
☐ Dark colour
☐ Blood admixture
☐ Mucus admixture

C. Health of other animals

20. Does any of your other animals have had diarrhea in the past month?

☐ Yes, namely (please specify which animal(s)):
....

☐ No
☐ No, I do not have any other animals

21. Does any of your other animals have been treated with medicines in the last year?

☐ Yes, namely (please specify which animal and which kind of medicine):
....

....

☐ No
☐ No, I do not have any other animals

Supplementary Materials Part F: Questionnaire Dog

II. II. General information

A. Administrative details veterinarian/veterinary practice

**Name + first name**		
**Address (personal)**	Street:	nr.:
	Postal code:	City:
	Country:	
**Telephone nr.**		
**Mobile phone nr.**		
**Email address**		

B. Administrative details dog owner

**Name + first name**		
**Address (personal)**	Street:	nr.:
	Postal code:	City:
	Country:	

**Description of residential area**	☐ Resident (mainly pavement and houses)<br>☐ Rural (many feedlot, little forestation)<br>☐ Wooded<br>☐ Other (please specify):………………………………………………………………………
**Telephone nr.**	
**Mobile phone nr.**	
**Email address:**	

General information Dog

**Name dog**	
**Breed dog**	
**Age**	
**Sex dog**	☐ Female dog<br>☐ Male dog
**Neutered/sterilized**	☐ Yes<br>☐ No

C. Contact with other animals

22. Does your dog have contact with other animals, including farm animals?

☐ Yes, (please specify which animals)

☐ No

23. How much time per week does your dog spend in the house or outside?

☐ Always in the house (100% inside)
☐ Mostly in the house , occasionally outside (80% inside, 20% outside)
☐ Often in the house, sometimes outside (60% inside, 40% outside)
☐ Half of the time in the house and half of the time outside (50% inside, 50% outside)
☐ Often outside, sometimes in the house (60% outside, 40% inside)
☐ Mostly outside, occasionally in the house (80% outside, 20% inside)
☐ Always outside (100% outside)

24. How many animals do you keep in house, including your dog?

☐ 1
☐ 2
☐ 3
☐ 4
☐ 5
☐ >5

25. Which animals do you keep in house?

26. Did your dog stay in a pension/animal shelter in the past year?

- ☐ Yes
  Period: From .... (dd/mm/yyyy) to .... (dd/mm/yyyy)

- ☐ No

27. Does your dog eat faeces from other animals?

- ☐ Yes
- ☐ No
- ☐ Unknown

28. Do you walk your dog on dog walking fields?

- ☐ Yes
- ☐ No

29. Does your dog participate in any of the following activities (multiple answers possible)?
- ☐ Off-leash with other dogs
- ☐ hunting
- ☐ Dog day care
- ☐ Dog park
- ☐ Obedience/agility/flyball
- ☐ Kennelled/hospitalized in the last six months
- ☐ Other:

30. Are farms with livestock situated within a radius of 500 meters of your home?

- ☐ Yes
- ☐ No (go to question 12)
- ☐ Unknown (go to question 12)

31. Which type(s) of farm(s) are located within a radius of 500 meters of your home?

....

32. Does the dog have access to farms with livestock?

☐ Yes

What type(s) of farm(s) ....

....

☐ No

☐ Unknown

33. Has your dog had access to any of the following water sources, either for drinking or swimming, in the last six months? (Tick multiple boxes if applicable)

☐ Lakes, rivers, creeks

☐ Water in ditches, puddles

☐ Toilet

☐ Other:

☐ No

☐ Unknown

D. Food

34. What does your dog eat? (Tick multiple boxes if applicable)

☐ Dry food (prepacked (commercial))

☐ Wet food (prepacked (commercial))

☐ Cooked non-commercial meat

☐ Defrozen raw meat

☐ Raw meat

☐ Vegetarian
☐ Food remaining from the table
☐ Other, please specify:

....

35. Have you provided your dog with any of the following treats (multiple answers possible)?
☐ Dried pig's ears
☐ Raw Bones
☐ Cooked Bones
☐ Store bought bones
☐ Rawhide chews
☐ Other pet treats:

E. Health

36. Is your dog currently healthy?

☐ Yes
☐ No: Please specify why you think your dog is ***not*** healthy:

....

....

....

37. Has your dog been treated with medicines in the last year?

Please ask your veterinarian for a printout of the medical records.

☐ Yes, please fill in the table below.

☐ No

Name of the medicine	Period of use<br>From (dd/mm/yyyy)<br>To (dd/mm/yyyy)	Frequency of use<br>Number of times per day	Duration of use<br>Number of days
1.			
2.			
3.			
4.			
5.			
6.			
7.			
8.			
9.			

10.			

38. Has your dog been hospitalized in the last six months?

☐ Yes
☐ No (go to question 20)

39. What was the reason for hospitalization?

....

40. During which period was the hospitalization?

From (dd/mm/yyyy) .... To (dd/mm/yyyy) ....

41. Do you have noticed something remarkable in the stool of your dog in the past three months?

☐ Yes
☐ No (go to question 22)

42. What have you noticed in the stool of your dog? (Tick multiple boxes if applicable)

☐ Reduced consistency
☐ Increased consistency
☐ Light colour
☐ Dark colour
☐ Blood admixture
☐ Mucus admixture

F. Health of other animals

43. Does any of your other animals have had diarrhea in the past month?

☐ Yes, namely (please specify which animal(s)):

....

☐ No

☐ No, I do not have any other animals

44. Does any of your other animals have been treated with medicines in the last year?

☐ Yes, namely (please specify which animal and which kind of medicines):

....

☐ No

☐ No, I do not have any other animals

Supplementary Materials Part G: Quantification of antimicrobial usage and Antimicrobial susceptibility testing

Quantification of antimicrobial usage

treatment incidence (TI) was calculated by using a simplified formula as necessary assumptions lead to identical factors in both nominator and denominator. Firstly, a standard weight was used in both nominator and denominator, equaling 19.1 kg for dogs and 4.1 kg for cats [1]. Secondly, the assumed administered dose (ADD) in the nominator was the same as the defined daily dose for companion animals (DDDca) in the denominator, and both were derived from the SPC values. The latter adjustment was necessary since the European Surveillance for Veterinary Antimicrobial Consumption committee has not yet defined standardized Defined Daily Doses (DDD) for dogs and cats, like the DDDvet values for food-producing animals, nor did data collection include the exact administered dose. The total duration of the treatment per animal was retrieved from the questionnaire. One parenteral antimicrobial was used off-label and the DDDca was derived from literature instead of from the SPC [2]. For topical products, only the total duration of treatment and the recommended dose according to the SPC was used for calculation. A single dose for eye ointment was defined as 10 mm (20 mg) per eye [3]. Topical treatments of eyes or ears were considered a treatment for both eyes and ears, respectively [1,2]. When relevant, a long-acting (LA) factor was used to represent the duration of activity for LA products. This was based on the recommended treatment interval mentioned in the SPC and equaled 2, 3 and 14 for the products Amoxicillin LA®, Duplocilline LA® and Convenia®, respectively. As AMU data were collected on individual animal level and covered the year previous to the sampling date, the period at risk for treatment was set equal to a full year (= 365 days).

Antimicrobial susceptibility testing

The epidemiological cut-off values (ECOFFs) to define wild-type *E. coli* were: ampicillin (AMP) ≤8 mg/L, cefotaxime (FOT) ≤0.25 mg/L, ceftazidime (TAZ) ≤0.5 mg/L, meropenem (MERO) ≤0.125 mg/L, ciprofloxacin (CIP) ≤0.064 mg/L, nalidixic acid (NAL) ≤16 mg/L, azithromycin (AZI) ≤16 mg/L, chloramphenicol (CHL) ≤16mg/L, colistin (COL) ≤2 mg/L, gentamicin (GEN) ≤2 mg/L, sulphamethoxazole (SMX) ≤64 mg/L, trimethoprim (TMP) ≤2 mg/L, tetracycline (TET) ≤8 mg/L, and tigecycline (TGC) ≤0.5 mg/L.[4]

References

1. Van Geijlswijk, I.; Alsters, S.; Schipper, L. Voorschrijven van antimicrobiële middelen in de gezelschapsdieren-praktijk. *Tijdschr. Diergeneeskd.* **2013**, *9*, 25–29.
2. Wiebe, V.J. *Drug Therapy for Infectious Diseases of the Dog and Cat*; Wiebe, V.J., Ed.; 1st ed.; Wiley and sons: Ames, IA, USA, 2015;
3. WHO Collaborating Centre for Drug Statistics Methodology. *Guidelines for ATC Classification and DDD Assignment 2018*; WHO Collaborating Centre for Drug Statistics Methodology: Oslo, Norway, 2017;
4. Kahlmeter, G.; Brown, D.F.J.; Goldstein, F.W.; Macgowan, A.P.; Mouton, J.W.; Österlund, A.; Rodloff, A.; Steinbakk, M.; Urbaskova, P.; Vatopoulos, A. European harmonization of MIC breakpoints for antimicrobial susceptibility testing of bacteria. *J. Antimicrob. Chemother.* **2003**, *52*, 145–148.
